# Sensorized Robotic Skin Based on Piezoresistive Sensor Fiber Composites Produced with Injection Molding of Liquid Silicone

**DOI:** 10.3390/polym13081226

**Published:** 2021-04-10

**Authors:** Antonia Georgopoulou, Silvain Michel, Frank Clemens

**Affiliations:** 1 Department of Functional Materials, Empa–Swiss Federal Laboratories for Materials Science and Technology, Überlandstrasse 129, 8600 Dübendorf, Switzerland; 2 Department of Mechanical Engineering (MECH), Vrije Universiteit Brussel (VUB), and Flanders Make Pleinlaan 2, B-1050 Brussels, Belgium; 3 Department of Engineering Sciences, Empa–Swiss Federal Laboratories for Materials Science and Technology, Überlandstrasse 129, 8600 Dübendorf, Switzerland; Silvain.Michel@empa.ch

**Keywords:** injection molding, fiber composites, electronic skin, stretchable electronics

## Abstract

Soft robotics and flexible electronics are rising in popularity and can be used in many applications. However, there is still a need for processing routes that allow the upscaling in production for functional soft robotic parts in an industrial scale. In this study, injection molding of liquid silicone is suggested as a fabrication method for sensorized robotic skin based on sensor fiber composites. Sensor fibers based on thermoplastic elastomers with two different shore hardness (50A and 70A) are combined with different silicone materials. A mathematical model is used to predict the mechanical load transfer from the silicone matrix to the fiber and shows that the matrix of the lowest shore hardness should not be combined with the stiffer fiber. The sensor fiber composites are fixed on a 3D printed robotic finger. The sensorized robotic skin based on the composite with the 50A fiber in combination with pre-straining gives good sensor performance as well as a large elasticity. It is proposed that a miss-match in the mechanical properties between fiber sensor and matrix should be avoided in order to achieve low drift and relaxation. These findings can be used as guidelines for material selection for future sensor integrated soft robotic systems.

## 1. Introduction

Injection molding is a common process for the shaping of thermoplastics [1,2,3], thermoplastic elastomers [4,5,6], composites [7,8,9], as well as metals [10,11,12] and ceramics [13,14]. The central concept around the method is the melting and plasticization of the material and feeding it into a mold cavity, where the material is solidified by cooling [15]. The process is completely industrialized and automatized for mass production [16]. The process can also be scaled down for prototype fabrication [17,18] and even the expensive mold fabrication can be scaled down using additive manufacturing methods [19,20,21,22].

In comparison to standard injection molding for thermoplastics, the liquid injection molding process, includes a pump that mixes A and B components of liquid silicone rubber (LSR) and injects it into a heated mold cavity, where the LSR cures [23,24]. Typically, component A contains a platinum catalyst and component B contains methylhydrogensiloxane as a cross-linker and an alcohol inhibitor [25,26]. Because of the thermosetting nature of the material, special care needs to be taken during injection molding, in limiting the intensity of the mixing and the temperature, to avoid unwanted vulcanization at the early stages of the process [27]. The curing of silicone rubber can be accelerated with increasing temperature, which will reduce the time of the injection molding cycle [28]. Additionally, the pressure in the cavity has to be taken into account, in order to achieve defect-free pieces. Injection molding of liquid silicones finds application in the field of soft electronics, especially for embedding sensitive electronic structures like optoelectronic components [29]. Embedding electronics in elastomers can protect the embedded part from environmental conditions or damages because of incorrect handling. In addition liquid silicone rubber is particularly interesting for the development of flexible electronics [30]. So far, liquid injection moulding has not been used for the fabrication of piezoresistive fibrous based sensor composite.

Sensorized robotic skin (namely tactile sensors) that can be used in robotics has already been investigated [31,32]. The sensorized robotic skin acts as an interface between the robot and its environment [33]. The tactile sensing can contribute to the better perception of the robot’s environment [34,35,36] and better human–robot interaction [37]. Especially in biomedical applications, soft robotic skin can contribute to the development of prosthetics with sensing abilities that can restore some of the sensitive functions of a lost limb [38,39,40]. Similar to biological skin, robotic skin must be elastic and must have the ability to endure large elongations. For that reason, elastomer materials like silicone rubber are excellent candidates for the development of sensorized robotic skin. To avoid stiffening of the resulting composite, Georgopoulou et al. have demonstrated that using the fiber composite concept, a higher stiffness of flexible elastomer strain sensor composites can be avoided [41,42]. Typically, these sensorized robotic skins are mainly produced by hand-made casting process. 

There is still a need for a fabrication method that is compatible with industrial manufacturing and can lead to part production with good reproducibility. Therefore, liquid injection molding is featured in this study, as a method to manufacture RTV-2 silicone composites with integrated piezoresistive sensor fibers. Injection molding is a processing method with great relevance for industrial production and it is assumed that, by this approach, flexible sensorized silicone structures for soft robotic skin, health monitoring and rehabilitation tools will come closer to industrial production. It is the first time that liquid injection molding is investigated for the fabrication of sensorized robotic skin (sensor fiber composite). It is necessary to investigate material combinations of the sensor matrix and the elastomeric fiber to achieve sufficient sensor properties (low drift and relaxation) of the sensorized robotic skin. Two different piezoresistive fiber sensors were embedded in three different silicone matrices with different shore hardness, using liquid injection molding process. The electromechanical properties of the six different fiber-matrix combinations were analyzed using tensile testing. The aim of the testing was to yield conclusions about the appropriate material selection, in terms of the mechanical properties and optimal sensor performance of the sensor fiber composites, for the injection molding of sensorized robotic skin. Finally, the concept of pre-straining, which was proposed by Georgopoulou et al. [42] was included for the sensorized robotic skin demonstrator on a robotic finger. The finger structure was made with an open source design and extrusion-based additive manufacturing. 

## 2. Materials and Methods

### 2.1. Fiber Sensor Preparation

For the piezoresistive fiber sensors, two styrene-ethylene/butadiene-styrene triblock copolymer (TPS) with shore hardness of 50A and 70A (Kraiburg TPE, Waldkraiburg, Germany) were mixed with carbon black (TIMCAL, Bodio, Switzerland). Both TPS and the carbon black were mixed in an equal mass ratio using a torque rheometer (Thermofisher, Karlsruhe, Germany). The fiber sensors were extruded using a capillary rheometer (NETZSCH-Gerätebau GmbH, Selb, Germany). The extruded fibers had a diameter of 0.5 mm. The fiber production is described in detail elsewhere [43,44]. In this paper, the two fibers will be referred to as ShA50 and ShA70.

### 2.2. Sensor Fiber Composite Preparation by Liquid Injection Molding of Silicone Rubber

RTV-2 silicone rubbers with a shore hardness of 25A, 40A, and 60A were obtained from Drever Otoplastik (Unna, Germany). The selected silicone had a high tear strength to ensure that the samples endure the demolding process.

**Figure 1:**
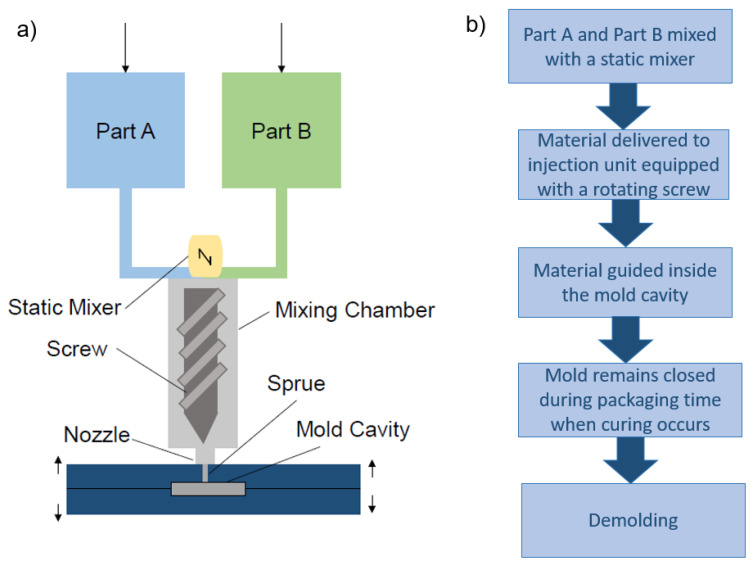
Sketch of (**a**) the basic parts of liquid injection molding machine, (**b**) the different processing steps for the fiber sensor fiber composite.

The liquid injection molding process was performed in three steps: First, a bottom layer was injected, in a second step the fiber sensor was placed on the bottom layer and a top layer was injected in a third step. For the liquid injection molding, a custom-made injection molding device equipped with a screw for homogeneous mixing was used (Figure 1).

As shown in Figure 1b the part A and B of the RTV-2 silicone materials were mixed before the injection step. It is worthwhile to mention, that due to the exothermal chemical reaction during the mixing, the material already heats up before reaching the mold cavity. Therefore, the duration time in the static mixer, screw, nozzle, sprue, runner, and gates must be limited. The mold was heated to 40 °C and curing time of 25 min with a backpressure of 4 bar was used.

### 2.3. Tensile Testing

In order to investigate the piezoresistive response of the sensor, the electrical resistance was recorded during tensile testing. A Zwick Roell Z005 tensile testing machine with a 200 N load cell and pneumatic clamps (4 bar) was used (ZwickRoell, Ulm, Germany). Tensile tests were performed up to failure of the sample, under dynamic (cyclical testing) and quasistatic conditions (cyclical testing with dwell time). For the electrical measurements, a Keithley 2450 multimeter from Keithley Instruments (Solon, OH, USA) was used. The relative resistance (*R_rel_*) was calculated using the following equation:

(1) $$R_{rel}=\frac{R-Ro}{Ro}$$

where *R* is the value of the resistance and *Ro* the initial resistance when no strain is applied to the integrated fiber sensor. To define the sensitivity of the sensor, the gauge factor (*GF*) was calculated by the following equation:

(2) $$GF=\;\frac{R_{rel}}{\Delta \varepsilon }$$

where Δ*ε* is the range of strain in which the *Rrel* is measured.

The electrical and mechanical relaxation were defined as a percentage from the dynamic and quasistatic cyclical tests, respectively. Georgopoulou et al. have reported these analysis methods for casted and 3D printed piezoresistive elastomer composites to select the final material combination, successfully [42,45]. The electrical drift is the difference of the electrical resistance between two dynamic cycles at a fixed strain value, and the relaxation is the difference of the electrical resistance at the beginning and the end of the dwell time during the quasistatic testing [44].

### 2.4. Modeling of the Stresses Exerted on the Fiber Inside the Composite

In order to calculate the stresses exerted on the fiber inside the composite, the engineering stresses were converted to true stresses using the following equation:

(3) $$\sigma_{true}=\sigma_{eng}(1\;+\;\frac{v}{0.5}\varepsilon \;)\;$$

where $\sigma_{true}$ is the true stress, $\sigma_{eng}$ is the engineering stress (the force divided by the cross-section area), v is the Poisson’s ratio and ε  is the strain for which the engineering stress was measured. The Poisson’s ratio for the silicone elastomer was considered to be 0.497 based on reports from previous studies [46,47,48]. As for the sensor fiber that was based on a thermoplastic elastomer and carbon black mixed in mass ratio 1:1, the Poisson’s ratio was assumed to be 0.315 based on observations from previous studies about carbon-filled composites [49,50].

According to the general rule of mixtures, the stresses in the composite can be calculated by the equation:

(4) $$\sigma_{tot}=\sigma_{F}\frac{A_{F}}{A_{tot}}+\sigma_{M}\frac{A_{M}}{A_{tot}}$$

where *σ_tot_* is the true stress exerted on the fiber composite, *σ_F_* is the true stress on the fiber inside the composite and *σ_M_* is true stress on the matrix, *A_F_* is the cross-section area of the fiber, *A_M_* is the cross-section area of the matrix of the composite, and *A_tot_* is the total cross-section area of the composite. The true stress on the fiber was obtained from the tensile test up to the point of fracture for the fiber. The true stress on the fiber embedded in the composite can be calculated, using Equation (5).

(5) $$\sigma_{F}=\sigma_{tot}\frac{A_{tot}}{A_{F}}-\sigma_{M}\frac{A_{M}}{A_{F}}$$

### 2.5. Robotic Finger with Sensorized Robotic Skin

**Figure 2:**
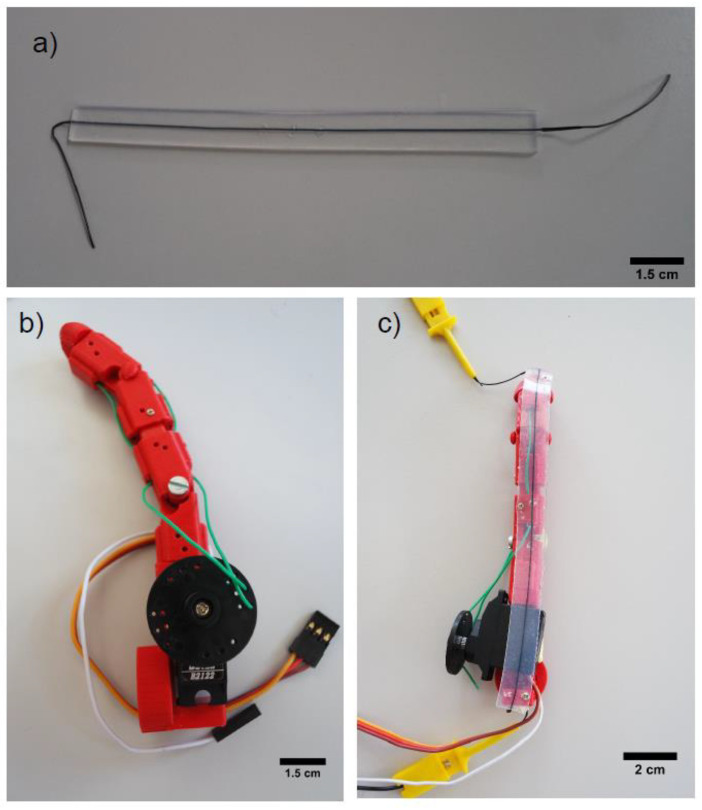
(**a**) Sensor fiber composite produced with liquid injection molding of silicone rubber; (**b**) design of the 3D printed robotic finger produced with 3D printing; (**c**) attached sensor fiber composite.

The robotic finger was produced by fused deposition modeling (FDM) technology, using polylactic acid (PLA) material. The design of the finger was open source [51] and it possessed modular parts and joints to make it flexible (Figure 2). For the actuation, flexible tendons connected with a servomotor actuator Batan B2122 (Adafruit, New York, NY, USA), driven by 6V and controlled by an Arduino microcontroller, were used. As sensorized robotic skin, the sensor fiber composite was attached on the finger with tapping screws. It was attached under pre-strain, since it was known from a previous study that pre-straining of such sensor fiber composites improve the linearity of the sensor signal at low strains [42]. The calculated maximum elongation of the sensor fiber composite on the robot finger was 50%. The electrical resistance signal of the sensor fiber composite while bending the finger was monitored by the Keithley 2450 multimeter (Keithley Instruments, Solon, OH, USA).

## 3. Results and Discussion

### 3.1. Tensile Tests up to Point of Fracture

**Figure 3:**
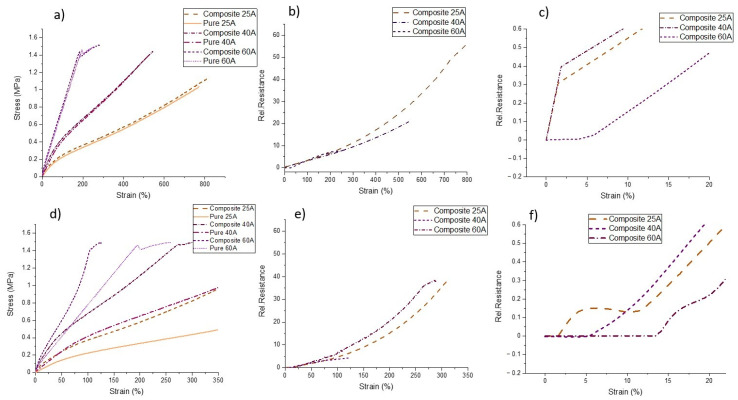
(**a**) Stress₋strain response for the fiber composites made with the Sh50A sensor fiber in a silicone matrix with shore hardness 25A, 40A and 60A and the pure matrix (**b**) electrical relative resistance-strain response and (**c**) zoom of the relative resistance-strain response between 0% and 20% strain (**d**) Stress₋strain response for the fiber composites made with the Sh70A sensor fiber in a silicone matrix with shore hardness 25A, 40A and 60A and the pure matrix (**e**) electrical relative resistance-strain response and (**f**) zoom of the relative resistance-strain response between 0% and 20% strain.

The integration of sensor fibers in soft materials is a strategy for developing functional electronic structures with sensing capabilities without increasing the stiffness of the soft material significantly. In order to assess the effect of adding the sensor fiber in the silicone rubber on the mechanical properties, a tensile test to the point of fracture was performed for the composite, but also for the pure silicone rubber (Figure 3).

In the case of the sensor fiber ShA50, the addition of the fiber didn’t affect the mechanical properties of silicone matrix significantly (Figure 3a). A small increase in the case of the 25A composite compared to the case of the 40A composite at low strains can be observed. For the case of the 60A composite and the 40A at high strains, the mechanical behavior of the composite and the pure polymer is almost identical. In general, using sensor fiber composites prevents significant stiffening of the elastomer. This effect can be unwanted for applications like soft robotics, where the elasticity of the material is an important requirement [52]. However, as can be seen from the case of the ShA70 fiber (Figure 3d), a higher stiffness of the fiber sensor results in a significant change in the stiffness of the composite. An important implication of the stiffening is the reduction of the working range of the sensor. In the case of the ShA50, the 25A composite had a working range of more than 800% strain, but for the ShA70 sensor, the same composite had a working range of only up to 350% strain.

Looking at the piezoresistive response of the fiber sensor embedded in the different silicone matrix materials, there can be seen that overall the relative resistance increases, by increasing the strain. In the case of the sensor fiber composites 40A and 60A with the ShA70 fiber, it can be seen that at low strains the relative resistance didn’t increase (Figure 3f). In the case of the ShA70 25A composite, the resistance first increased, then slightly decreased and after a strain of 13% it finally increased until failure. For the ShA50 fiber, the sensor fiber composites with 25A and 40A show similar behavior: At low strains, a sharp increase in resistivity was observed. For the 60A composite, the relative resistance slowly increases at low strains (Figure 3c). In Table 1, the values for the gauge factor (*GF*) for different strain ranges are listed. Up to a strain of 150%, the *GF* was almost the same for all fiber sensors embedded in the different silicone matrices. It can be assumed that the shore hardness of the silicone material does not affect the sensitivity of the fiber sensor in this strain range. The ShA70 sensor fiber composites exhibit a slightly higher sensitivity, expressed by a larger gauge factor. This observation is in good agreement with a previous work by Georgopoulou et al. Therein, it was reported that for stiffer matrix materials, higher sensitivity can be achieved [41]. It can be seen in Table 1 from the values of the initial resistance that for composites made with the two different fibers, with increasing the shore hardness of the matrix, the conductivity increased. The small deviation that was observed for the initial resistance can be traced to the fact that the fibers were manually placed in the middle of the matrix during the manufacturing process. In comparison to sensor fiber composites fabricated by mold casting, the deviation of the initial resistance was smaller. This is a good indication that, by injection molding, higher reproducibility in sample production can be achieved [42].

### 3.2. Dynamic Tensile Tests

**Figure 4:**
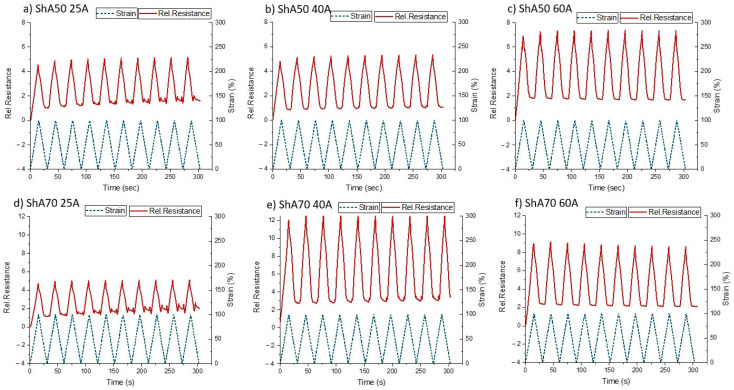
Relative resistance during dynamic cyclic tensile testing (10 cycles) between strains 0100%, for the sensor fiber composites with the fiber ShA50 embedded into the three different silicone materials (**a**) 25A (**b**) 40A (**c**) 60A and of the fiber ShA70 embedded into three different silicone materials (**d**) 25A (**e**) 40A (**f**) 60A.

The sensitivity and the effect of pre-straining were investigated by dynamic tensile tests. As it can be seen in Figure 4, the relative resistance of the sensor fiber composites was measured over ten cycles of straining and releasing. Based on the almost linear behavior between a strain of 20 and 150% (constant *GF* in Table 1) during the tensile testing up to the point of fracture, was selected between 0–100%.

From the dynamic tensile testing, it was seen that for all fiber sensors during the loading, the relative resistance followed the change in strain. However, for both composites based on 25A silicone material in the releasing phase of the cycle, there are two secondary peaks in the electrical sensor signal (Figure 4a,d). These secondary peaks appeared for both composites below a strain of 48%. Therefore, values of relative resistance in this range correspond to more than one strain values, which is not suitable for low-strain applications. Two secondary peaks can also be observed after the fifth cycle in the case of the Sh70 fiber in the 40A matrix (Figure 4e). Looking at the composites with higher shore hardness (Figure 4c,f) no secondary peaks appeared during the releasing. However, a plateau in the electrical response of the sensor fiber composites was detected at low strains. The appearance of the plateau and the secondary peaks attribute uncertainty to the sensor response. They could be caused by buckling of the fiber in the releasing part of the cycle. Buckling is described as the phenomenon whereby the sensor fiber composites do not return to the original length after the load is removed. These phenomena can be linked with the viscoelastic behavior of polymers. The fact that the stress became zero or even negative can be used to identify the range of strains where the buckling occurred during cycle testing.

In order to assess the effect of the buckling, the strain where the stress became zero was identified (Supplementary Figure S1) and compared with the range of the uncertainty of the sensor signal for the different sensor fiber composites. The values can be found in Table 2. The uncertainty of the sensor signal response was identified as the range of strains where plateau or secondary peaks appeared.

From Table 2, it can be seen that the range of strains where the buckling and the uncertainty occurred are different. Nonetheless, the strain values in Table 2 do not correlate with the lagging phase observed during the tensile test up to the point of fracture (Figure 3c,f). For the case of the composites with the softest matrix, the uncertainty appeared at a higher strain than the buckling. The buckling can be linked with the plateau for the ShA70 40A, ShA70 60A, and the ShA50 60A, but it still can’t explain the appearance of the secondary peaks for the composites of lower shore hardness. In addition, the fact that the secondary peaks appeared in the case of the ShA70 40A but not for the ShA50 40A composite is not explained with this interpretation of the data.

**Figure 5:**
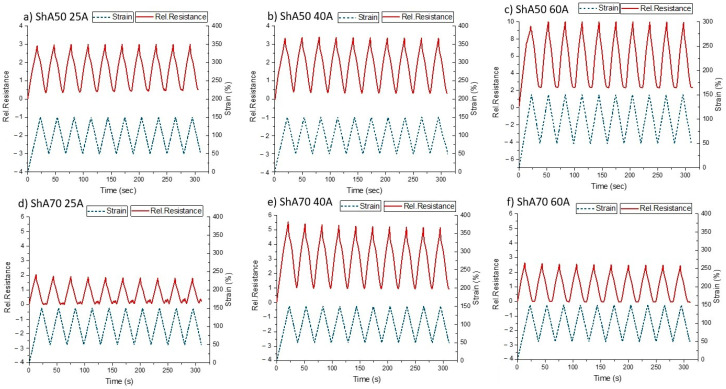
Change of relative resistance during dynamic tensile testing for the sensor fiber composites with the fiber ShA50 and three different matrix materials (**a**) 25A (**b**) 40A (**c**) 60A and of the fiber ShA70 embedded in a matrix of shore hardness (**d**) 25A (**e**) 40A (**f**) 60A. The sensors were strained in ten cycles, between strains 50–150%.

Effects of stress relaxation that cause the buckling behavior during the testing are well known for elastomers and are connected with the viscoelasticity of the elastomer materials [53]. In order to cope with these effects and the uncertainty they cause on the sensor response, an engineering solution is to pre-strain the sensor, in order to use the sensors above the strain where buckling appeared. It is assumed, that the same strategy can be used for the sensorized robotic skin application on the robotic finger. According to Table 1, the *GF* does not significantly change up to a strain of 150%. Taken the strain into account where buckling and uncertainty of the sensor signal occur (Table 2), dynamic testing was repeated between 50% and 150% strain (Figure 5).

For the dynamic testing, the drift of the sensor signal was calculated at 100% strain between the second and tenth cycle (Table 2). The drift was calculated at 50% strain and 100% strain. For all of the composites, the drift was lower at higher strains. There was not a significant difference observed between the composites with the ShA50 fiber and the ShA70 fiber. The composites with the lower shore hardness (25A) exhibited the higher drift at 50% strain. This can be explained by the fact that 50% is very close to the uncertainty area range of the sensor signal. For the other sensor fiber composites, the low drift, especially at high strains can be a significant advantage for the use of the sensors in several applications.

From the dynamic tensile testing at 50–150%, it can be seen that for the composites with the ShA50 fiber (Figure 5a–c) the response is linear in both the loading and the releasing, even for the 25A shore hardness composite. The values of the stress–strain curve confirm that no buckling occurred at this area of strain (Supplementary Figure S2). When looking on the response signal of the 25A composite with ShA70 fiber, it can be seen that the secondary peaks in the unloading is still present.

### 3.3. Modeling of the Stress-Strain Behavior of the Sensor Fiber Embedded in the Matrix

**Figure 6:**
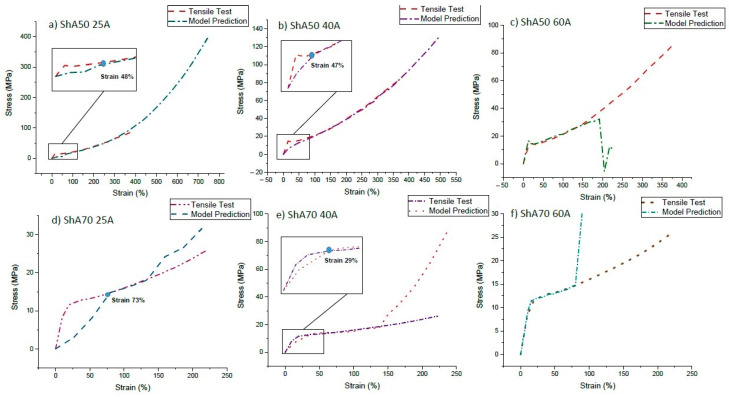
Stress₋strain curve of the single sensor fibers measured by tensile testing and calculated from the measurements of the composites using equation 5. Experimental and modeled curves for Fiber ShA50 and different silicone matrix materials (**a**) 25A (**b**) 40A (**c**) 60A. Experimental and modeled curves for Fiber ShA70 and different silicone matrix materials (**d**) 25A (**e**) 40A (**f**) 60A.

Even though pre-straining the sensors was a successful strategy to avoid the buckling of the composite in the unloading phase during the cycle testing, for the ShA70 25A sensor composite, secondary peaks still appeared. It was assumed that insufficient load transfer between the fiber and the matrix could be the reason why secondary peaks still can be observed. In Figure 6, the measured and the modeled predicted stress–strain curve of single sensor fibers are shown. The values predicted by the model are calculated using the measurement of the sensor fiber composites. The model prediction for the stress–strain curves were calculated using Equation (5).

It can be seen that the stresses predicted by the model correspond well with the tensile measurements using a silicone matrix material with high shore hardness of 60A. However, in the case of the ShA50 25A, ShA50 40A, and ShA70 40A composites, a deviation between the predicted stress and directly measured on the sensor fiber was observed. For the composites with a 40A matrix and the ShA50 25A fiber composite, the strain range, where this deviation happens, corresponds to the strain range where the uncertainty was defined in Table 2. Based on the assumptions of the model, it can be assumed that insufficient or non-uniform load transfer into the fiber at low strain occurs. At low strains, there is no strong bonding between fiber and matrix and therefore the model is not valid at low strains. At high strains, a deviation between predicted and measured stresses can also be observed (Figure 6). This can be explained by the different plastification of the two materials and a delamination effect.

For the ShA70 25A composite, predicted and measured stresses differ significantly up to a strain of 75%. Interestingly, in the dynamic experiments with the pre-straining, the same composite still shows a secondary peak. This is in good agreement with our assumption that load transfer is not uniform in the case of the ShA70 25A composite. The composite model used in this study helped to understand why the sensor signal at low strains results in uncertainty. As shown in Figure 5, the pre-straining approach above the strain range where predicted and measured stresses are different improved the sensor signal behavior.

### 3.4. Effect of Relaxation

**Figure 7:**
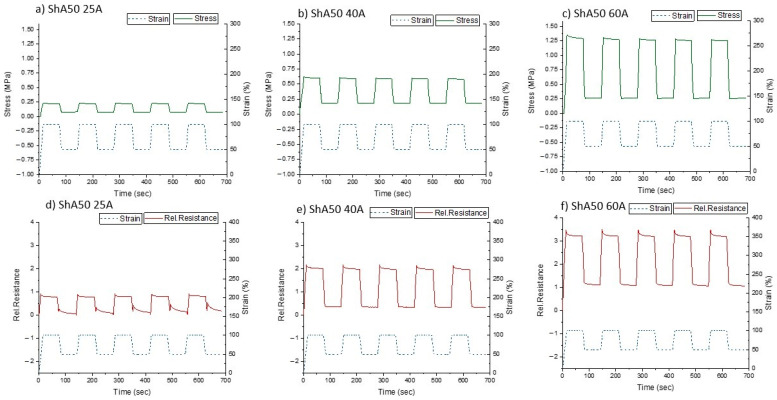
Quasistatic cycle tests for ShA50 fiber embedded in silicone matrix with different shore hardness; Upper line: Mechanical response with matrix type (**a**) 25A (**b**) 40A (**c**) 60A; Lower line: Electrical response with matrix type (**d**) 25A (**e**) 40A (**f**) 60A. The quasistatic tensile testing was between 50% and 100% strain. The testing involves a dwell time of 60 s at 50% and 100% strain.

In order to estimate the effect of the mechanical relaxation and the relaxation of the electrical signal on the sensor fiber composites, a cyclic tensile test with a dwell time was performed (quasistatic cycle testing) [52]. Based on the pre-straining results and the later application (soft robotic skin with maximal strain of 50%), the relaxation behavior of the ShA50 sensor fiber composites was tested in the strain range of 50–100% with a dwell time of one minute (Figure 7).

As expected, the relative resistance followed the change in strain. Due to the positive stresses during the testing, it can be assumed that buckling could be avoided for all samples. The relaxation for the mechanical (stress) and electrical signals (relative resistance) are shown in Table 3 and Table 4, respectively.

The electrical relaxation was higher than the mechanical relaxation. The electrical relaxation was higher at lower strains. The composite with the 25A matrix showed the highest relaxation of the electrical signal. Especially at low strains, the large amount of signal relaxation related to the appearance of a secondary peak was linked with the buckling of the elastomer matrix.

### 3.5. Application: Sensorized Robotic Skin for Robotic Finger

**Figure 8:**
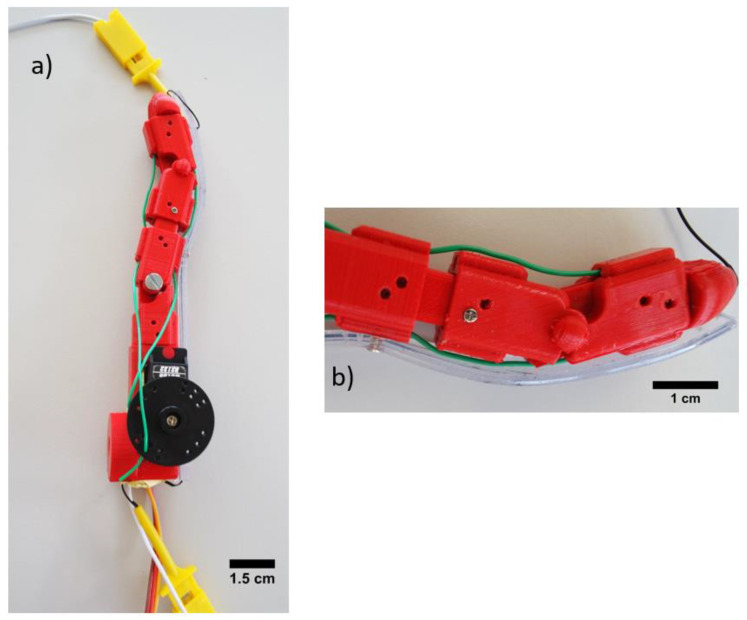
(**a**) Robotic finger with attached sensor fiber composite based skin and (**b**) To stabilize the skin on the finger structure during movement, the sensorized skin was fixed in the middle of the strip with an additional screw.

In order to show the potential of the sensor fiber composites produced by injection molding, the sensor fiber composites were fixed on the robotic finger with a pre-strain of 50% (Figure 8). The robotic finger was equipped with a servo motor and tendons to allow the movement of the joints. The sensor signal of the ShA50 fiber monitored the bending of the robotic finger.

**Figure 9:**
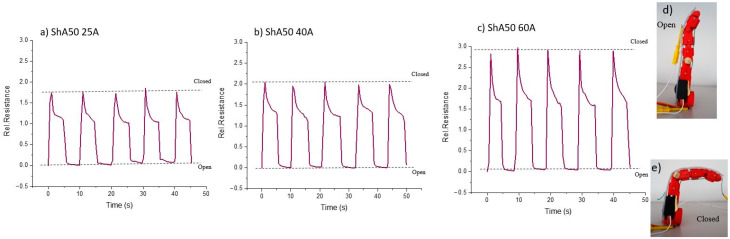
Electrical response of the piezoelectric fiber composites fixed on a robotic finger during finger bending. The electrical signals for the composites with shore hardness (**a**) 25A (**b**) 40A and (**c**) 60A are shown for a quasistatic test (5 s dwell time). The finger moved to position (**d**) open from position (**e**) closed.

Looking at the results in Figure 9, it can be seen that the piezoresistive skin could recognize the bending of the robot finger (open and closed) in a repeatable way (Figure 9a–c). The full bending of the finger (closed versus open) resulted in a 50% strain of the sensorized skin. The dotted lines show that during the five cycles no significant drift was observed.

The sensitivity of the composites decreases with the shore hardness of the matrix in a similar way as in the quasistatic tensile tests with pre-straining (Figure 7). However, a significant relaxation of the electrical signal can be observed during the dwell time, when the finger is closed. This could not be observed during the quasistatic tensile tests. A possible explanation for the different behavior could be that the motion of the finger caused not only uniaxial elongation in the skin, but also bending. Furthermore, the sensor covers two hinges that are not uniformly bent. Therefore, it can be assumed, that the design of the robot finger and the fixation of the sensorized skin affect the sensor response and performance. However, even with the large electrical relaxation, the sensorized skin can indicate the end position of the finger (open and closed). Further investigation is needed to improve the robot design and mounting method of the sensitive skin to reduce the relaxation of the electrical signal in order to increase the precision of the monitoring of the finger bending.

## 4. Conclusions

In this study, sensor fiber composites were produced using liquid injection molding of RTV-2 silicone rubber and piezoresistive sensor fibers. Silicone rubber of different shore hardness (25A, 40A and 60A) was used. The injection molding was performed in three steps. The first step included injection of a silicone layer. In the second step, the fiber was placed on top of the layer. Finally, an upper layer of silicone was injected on top of the fiber. Piezoresistive sensor fibers with two different values of shore hardness (50A and 70A) were investigated. It was demonstrated that stiffening of the composite could be avoided using the softer sensor fiber (ShA50). Secondary peaks in the sensor signal, during unloading at low strains could be observed during dynamic tensile cycles. However, by using the pre-straining approach, the secondary peaks could be avoided in almost all composites. The fiber composite model could be used to better understand the sensor behavior in the pre-strained configuration. It was observed that the appearance of the secondary peaks correlated with a difference of the calculated and measured stress–strain curves. Based on these results, it was assumed that insufficient or non-uniform load transfer between fiber and matrix occurred at low strains. The assumption of ideal load transfer in the model was violated at these strains and stress shielding occurred in the silicone matrix with low shore hardness.

Sensorized skin, based on the piezoresistive sensor fiber composites was attached on a robotic finger. The electrical response showed good repeatability, no significant drift in the sensor signal and higher sensitivity for composites with higher shore hardness matrix. However, the relaxation of the sensor signal in the sensorized skin was significantly higher than expected from the quasistatic tensile measurements. This difference in the behavior of the skin mounted on the robot finger compared to the results of the skin in the tensile tests can be explained with the design of the robotic finger and the fixation of the skin on the finger. While in tensile tests, the skin is only elongated uniaxialy, mounted on the robot finger, the skin is elongated and bent. Nevertheless, the sensorized skin realized here can already detect finger movements in general, and in particular when the finger is in an “open” or “closed” position. In the future, the bending behavior of such composites should be investigated. In that case, a better correlation of the sensor signal between test experiments and real robotic application can be expected.

Overall, liquid injection molding of silicone rubber can be used for the fabrication of sensor fiber composites. Targeted selection of silicone rubber for the matrix and the sensor fiber in combination with pre-straining are needed to achieve reliable signal behavior of the sensor fiber composite as a sensitive skin with low drift and low relaxation. This study demonstrates that industrial production processes can be used for the development of functional soft robotic materials by simple downscaling. It can be assumed that this technology will help to build up soft robotic structures with integrated sensors in a more robust and less time-consuming process. The compatibility with industrial practices will help to fabricate functional soft robotic components like sensorized skin in the near future.

## Figures and Tables

**Table 1:** Values of the initial resistance (*Ro*) and the electrical gauge factor calculated at different strain ranges during the tensile test to the point of fracture for the sensor fiber composites, with sensor fibers in two different shore hardness (ShA50 and ShA70).

Composite	R0 (kΩ)	GF (ε = 20–100%)	GF (ε = 100–150%)	GF (ε = 150–200%)	GF (ε = 200–270%)	GF (ε > 270%)
ShA50 25A	5.6 ± 0.6	3	3	3	6	6
ShA50 40A	4.6 ± 0.3	3	3	3	5	5
ShA50 60A	2.7 ± 0.5	3	3	3	2	-
ShA70 25A	6.5 ± 0.3	4	4	11	24	-
ShA70 40A	4.0 ± 0.8	5	5	14	24	-
ShA70 60A	2.7 ± 0.2	4	3	-	-	-

**Table 2:** Values for the drift calculated at strain 50% and 100% and the strain value where mechanical buckling (stress zero or negative) and uncertainty of the sensor signal appears during cycle testing between 0–100% strains.

Composite	Drift at 50% Strain (%)	Drift at 100% Strain (%)	Strain Where Buckling Appears (%)	Strain Where the Uncertainty of the Sensor Signal Appears (%)
ShA50 25A	23	1	17	49
ShA50 40A	11	1	20	47
ShA50 60A	13	5	32	36
ShA70 25A	23	3	27	47
ShA70 40A	14	1	28	21
ShA70 60A	8	5	32	35

**Table 3:** Stress relaxation of the ShA50 sensor fiber composites based on different silicone matrix materials. The quasistatic tensile testing was performed between 50% and 100% strain. The testing involves a dwell time of 60 s at 50% and 100% strain.

Shore Hardness of the Matrix	50% Strain	100% Strain
25A	4%	3%
40A	4%	2%
60A	13%	3%

**Table 4:** Relaxation of the electrical signal of the ShA50 sensor fiber composites based on different silicone matrix materials. The quasistatic tensile testing was performed between 50% and 100% strain. The testing involves a dwell time of 60 s at 50% and 100% strain.

Shore Hardness of the Matrix	50% Strain	100% Strain
25A	114%	13%
40A	9%	9%
60A	14%	8%

## Data Availability

No data other than that shown in the manuscript and in Supplementary Materials has been reported.

## Supplementary Materials

The following are available online at https://www.mdpi.com/article/10.3390/polym13081226/s1, Figure S1 and Figure S2.
