# Supplementary material for: Sensorized Robotic Skin Based on Piezoresistive Sensor Fiber Composites Produced with Injection Molding of Liquid Silicone
Source: Polymers (Basel). 2021 Apr 10;13(8):1226. doi: 10.3390/polym13081226 (PMC8070002; doi:10.3390/polym13081226)
Supplement: Supplementary file 1 [file polymers-13-01226-s001.pdf]

Supplementary Materials

Sensorized robotic skin based on piezoresistive sensor fiber composites produced with injection molding of liquid silicone

Antonia Georgopoulou*, Silvain Michel, Frank Clemens*

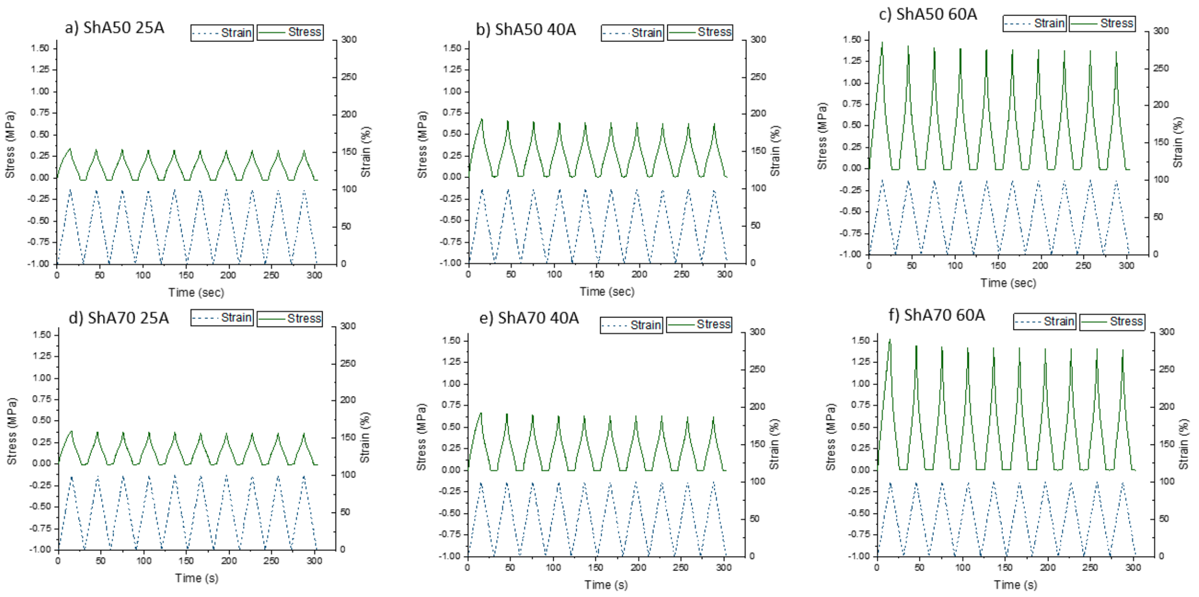

Figure S1. Mechanical response during dynamic tensile testing for the sensor fiber composites with the fiber ShA50 embedded in an elastomer matrix with shore hardness a)25A b) 40A c) 60A and of the fiber ShA70 embedded in a matrix of shore hardness d)25A e) 40A f) 60A. The sensors were strained in ten cycles, between strains 0-100%.

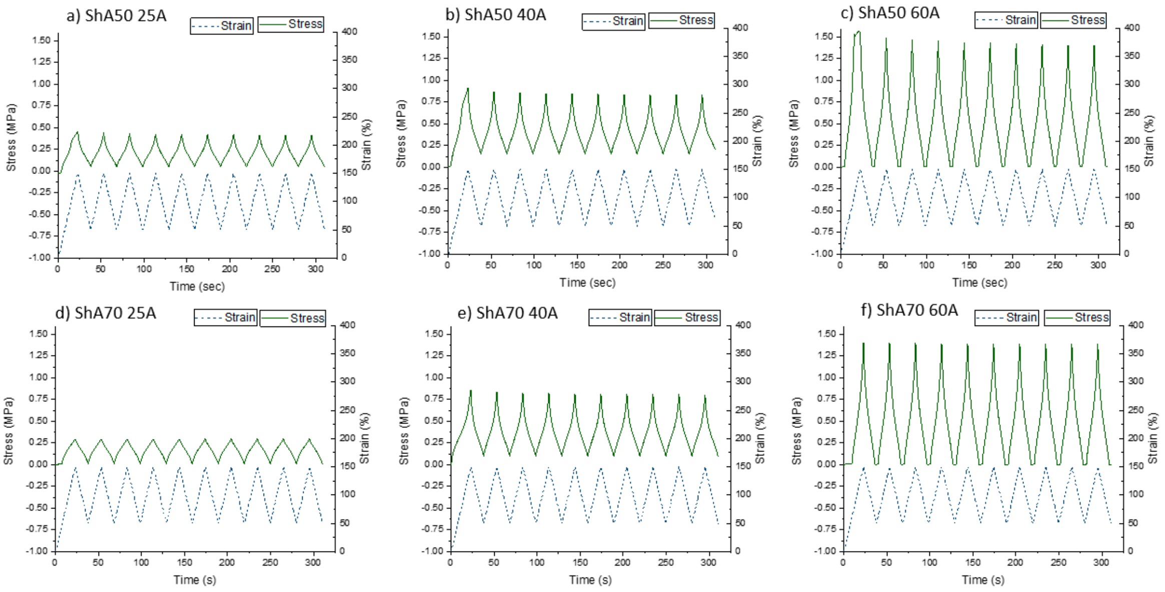

Figure S2. Mechanical response during dynamic tensile testing for the sensor fiber composites with the fiber ShA50 embedded in an elastomer matrix with shore hardness a)25A b) 40A c) 60A and of the fiber ShA70 embedded in a matrix of shore hardness d)25A e) 40A f) 60A. The sensors were strained in ten cycles, between strains 50-150%.
